# Antibacterial activity of crude extracts of some South African medicinal plants against multidrug resistant etiological agents of diarrhoea

**DOI:** 10.1186/s12906-017-1802-4

**Published:** 2017-06-19

**Authors:** Mary A. Bisi-Johnson, Chikwelu L. Obi, Babatunde B Samuel, Jacobus N. Eloff, Anthony I. Okoh

**Affiliations:** 1 Department of Microbiology, Obafemi Awolowo University, Ile-Ife, Nigeria; 2 Department of Medical Microbiology, Walter Sisulu University, Mthatha, 5117 South Africa; 3 Directorate of Academic Affairs, University of Fort Hare, Alice, 5700 South Africa; 4 Phytomedicine Programme, Department of Paraclinical Science, Faculty of Veterinary Science, University of Pretoria, Onderstepoort, 0110 South Africa; 5 SAMRC Microbial Water Quality, Monitoring Centre, University of Fort Hare, Alice, South Africa

**Keywords:** Medicinal plants, Diarrhoea, Antibacterial, Bioautography, Phytochemical

## Abstract

**Background:**

This study evaluated the antibacterial activity of some plants used in folklore medicine to treat diarrhoea in the Eastern Cape Province, South Africa.

**Methods:**

The acetone extracts of *Acacia mearnsii* De Wild.*, Aloe arborescens* Mill., *A. striata* Haw.*, Cyathula uncinulata* (Schrad.) Schinz*, Eucomis autumnalis* (Mill.) Chitt., *E. comosa* (Houtt.) Wehrh., *Hermbstaedtia odorata* (Burch. ex Moq.) T.Cooke*, Hydnora africana* Thunb*, Hypoxis latifolia* Wight*, Pelargonium sidoides* DC*, Psidium guajava* L and *Schizocarphus nervosus* (Burch.) van der Merwe were screened against *Staphylococcus aureus, Escherichia coli, Enterococcus faecalis*, multi-resistant *Salmonella enterica* serovar Isangi, *S. typhi, S. enterica* serovar Typhimurium, *Shigella flexneri* type 1b and *Sh. sonnei* phase II. A qualitative phytochemical screening of the plants extracts was by thin layer chromatography. Plants extracts were screened for antibacterial activity using serial dilution microplate technique and bioautography.

**Results:**

The TLC fingerprint indicated the presence of terpenoids and flavonoids in the herbs. Most of the tested organisms were sensitive to the crude acetone extracts with minimum inhibitory concentration (MIC) values ranging from 0.018–2.5 mg/mℓ. Extracts of *A. striata, C. uncinulata, E. autumnalis* and *P. guajava* were more active against enteropathogens. *S. aureus* and *Sh. flexneri* were the most sensitive isolates to the crude extracts but of significance is the antibacterial activity of *A. arborescens* and *P. guajava* against a confirmed extended spectrum betalactamase positive *S. enterica serovar* Typhimurium.

**Conclusion:**

The presence of bioactive compounds and the antibacterial activity of some of the selected herbs against multidrug resistant enteric agents corroborate assertions by traditional healers on their efficacies.

## Background

Gastrointestinal and enteric diseases remain a problem worldwide with the greatest burden of diseases in the sub-Saharan Africa and Asia [1, 2].These diseases cause nearly 19% of the 10 million worldwide deaths of children younger than 5 years old [3]. The diseases continue to be important causes of morbidity and incur substantial health-care costs [4, 5]. Severe acute bacterial gastroenteritis is caused majorly by *Shigella*, but *Salmonella* spp., some *E. coli* pathotypes, *Campylobacter* and *Vibrio* spp. play an important role in the epidemiology of diarrhoea, especially in certain areas of the globe [6, 7].

Treatment failures of enteric diseases, particularly, the emerging multidrug resistant enteric bacteria is a big challenge. Multiple antibiotic resistance is on the increase among clinical isolates of bacteria [8, 9] and the emergence of resistance to the 3rd and 4th generation beta-lactam drugs has complicated therapy. The burden of resistance to extended-spectrum cephalosporin and other beta-lactam drugs among the *Enterobacteriaceae* is enormous both in the hospital and community [10, 11]. Pharmaceutical industries have produced a number of new drugs in the last three decades. An estimated 122 drugs from 94 plant species active against other diseases have been discovered through ethnobotanical leads [12, 13]. Some of these include Ephedrine (bronchodilator) derived from *Ephedra sinica,* Quinine (antimalarial) from *Cinchona ledgerian* [14], the antimalarial compound Artemisin, derived from *Artemisia annua* L [15] and several antitumor compounds [16, 17]. Despite these big strides, resistance to antimicrobials by microorganisms is still on the increase. This is due mainly to the remarkable genetic plasticity of the microorganisms [18], inappropriate use, high selective pressures of use or under-use through inaccessibility, poor quality drugs, inadequate dosing, poor patient compliance and the increased mobility of the world population [19]. The incorporation of antimicrobials as growth promoting additives in animal feed is also a contributory factor to the emergence of drug-resistant bacteria [20, 21]. The sub-therapeutic use leads to bacterial exposure to sublethal concentration of drugs over a period of time leading to selection of resistance strains [22].

The rate at which new drugs are developed is not keeping pace with the changing virulence and drug resistant patterns of microbes. According to Kunin’s [18] review of the book by Owen and Lautenbach [23], antimicrobial resistance is the inevitable result of Darwinian evolution — natural selection and survival of the fittest. While there is a continuous modification of strategies by microbes in the ‘no victor no vanquish’ fight for survival, developing resistance to antibiotics is outpacing the pharmaceutical industry’s ability to develop new ones [24]. Drug resistance and development according to Ridley [25] takes 10 to 15 years and hence the quest for new drugs should be a continuous process [26, 27].

Medicinal plants have been acknowledged as potential sources of new compounds of therapeutic value and as sources of lead compounds for drug design and development [13, 28]. Various studies have documented the use of medicinal plants in various parts of the world including developed countries [27, 29, 30]. In contrast to other diseases, no antimicrobial with the potential for economic use has yet been discovered from plants. There are several possible reasons for this situation [31]. In South Africa, a sizeable number of both the rural and urban dwellers rely on traditional medicine for their primary health care [32, 33]. Some of the documented use of plants in stomach related ailments includes that of Iwu [34] in which immature fruits of *Olea europaea subsp. Africana* (Wild Olive), was reported to be used as astringents against diarrhoea. *Pentanisia prunelloides* (Klotzsch ex Eckl. & Zeyh) Walp is being used for a range of ailments and the root serves as enema for stomach pain [35]. However, many plants are yet to be explored scientifically and moreover, the need to find a lasting solution to the problem of infectious diseases with lingering treatment failures necessitated further exploration of natural products to uncover new grounds in drug production. This study screened and evaluated selected medicinal plants used in ethnomedicine in the Oliver R. Tambo municipality of Eastern Cape South Africa for their antibacterial activities against some enteropathogenic bacteria.

## Methods

### Plant material

Various plants parts used in the treatment of diarrhoea and related diseases in Oliver R. Tambo District municipality in the Eastern Cape province of South Africa were collected. These were *Acacia mearnsii* De Wild. (AC)*, Aloe arborescens* Mill., (AA), *Aloe striata* Haw. (AS)*, Cyathula uncinulata* (Schrad.) Schinz (CU)*, Eucomis autumnalis* (Mill.) Chitt*.* (E1 & E3)*, E. comosa* (Houtt.) Wehrh*.* (E2), *Hermbstaedtia odorata* (Burch. ex Moq.) T.Cooke (MB)*, Hydnora africana* Thunb. (UM)*, Hypoxis latifolia* Wight (HY)*, Pelargonium sidoides* DC. (PE)*, Psidium guajava* L. (PS)*, Schizocarphus nervosus* (Burch.) van der Merwe, (SC)*.* Samples of the plants were identified by the Kei Herbarium curator, Dr. Immelman, Walter Sisulu University, South Africa and voucher specimens deposited were tagged (Jaca 10 to Jaca 21). The traditional uses and the common names of the plants species selected are presented in Table 1.

**Table 1:** Names, plant parts and Traditional usage of herbs investigated

Plant Name	Common name	Plant part	Traditional usage	References
*Acacia mearnsii* De Wild. Family: Leguminosae	Blackwood Black Wattle	Bark	Diarrhoea, dysentery, sore throat, coughs, children fever, tooth ache	[35]
*Aloe arborescens* Family: Xanthorrhoeaceae	Aloe	Leaves	Vomiting, Skin ailments, diarrhoea, urinary complaints, rheumatism, tuberculosis	[35, 62]
*Aloe striata* Family: Xanthorrhoeaceae	Aloe	Leaves	Treatment of constipation	[63]
*Cyathula uncinulata* (Schrad.) Schinz Family: Amaranthaceae	NA	Leaves	Antidiarrhoea, philter or medicine for love	[64]
*Eucomis autumnalis (Mill.) Chitt.* Family: Asparagaceae	Common Pineapple Flower	Bulb	Decoctions of bulb and roots for colic, flatulence	[65]
*Eucomis comosa (Houtt.) Wehrh.* Family: Asparagaceae	Pineapple lily	Bulb	Help teething in children and to treat rheumatism	[66]
*Hermbstaedtia odorata* Wild Cockscomb Family: Amaranthaceae	Rooi-aarbossie	Leaves	Cleansing stomach wash alone or with *Acaccia xanthophloea* and *Cappa*	[35]
*Hydnora africana* Thunb. Family: Hydnoraceae	Warty Jackal Food, Jakkalskos Kanip	Tuber	Diarrhoea, plant dried ground raw for dysentery, amenorrhoea, swollen glands	[66]
*Hypoxis colchicifolia* Baker. *Family:* Hypoxidaceae	African potato	Tuber	Headaches, dizziness, mental disorders, to treat cancers, inflammation, HIV, diarrhoea	[67, 68]
*Pelargonium sidoides* DC. Family: Geraniaceae	Rose-scented Pelargonium	Root	Gonorrhoea, diarrhoea, dysentery, root decoction severe diarrhoea, stomach ailment in children	[35]
*Psidium guajava* L. Family: Myrtaceae	Guava	Leaves	Leaves used for diarrhoea, Infusion of leaves for bloody diarrhoea, infusion as enema for severe diarrhoea	[35]
*Schizocarphus nervosus* (Burch.) van der Merwe Family: Asparagaceae	White Scilla	Corms	Rheumatic fever, dysentery. All purpose herb.	[69, 70]

Key: *NA* not available

### Plant preparation and extraction

Plant parts were washed with distilled water, air-dried and milled into a fine powder with a Wiley Grinder. Fifty gram portion of ground dried material was soaked overnight in 500 ml of acetone on an orbital shaker. The samples were suction-filtered through a Whattman No 1 filter paper using a Buchner funnel. The extract was concentrated at 45 °C using a Rotavapor (Eyela N-1100, Rikakikai, China). The concentrated extract was transferred to a pre-weighed glass vial and allowed to dry at room temperature under a stream of air. Working stock solutions of extracts were obtained by re-dissolving in acetone to yield 10 mg/ml solutions. Plant materials were kept in air-tight containers while extracts were kept at 4 °C in the dark for further analysis.

### Phytochemical analysis of extracts

Thin layer chromatography (TLC) plates (Merck, silica gel 60 F_254_) were used to separate the extracts into chemical constituents. The TLC plates were prepared in duplicate and developed under saturated conditions in different mobile solvent systems according to Kotze and Eloff [36]: Ethylacetate: methanol: water (EMW) (40:5.4:4) polar/neutral; Chloroform: ethylacetate: formic acid (CEF) (20:16:4) intermediate polarity/acidic and Benzene: ethanol: ammonium hydroxide (BEA) (36:4:0.4) non-polar/basic. An aliquot of 10 μℓ of extract (representing 100 μg of the extract) was placed in a line c, 0.8 cm long and was separated by TLC using Merck, Kieselgel 60 F_254_ TLC plates in a closed, saturated TLC tank. For the qualitative evaluation of a given substance, the R_f_ value (retention factor) of chromatograms was used as the parameter for comparison. The R_f_ value of a substance is the ratio of the distance moved by the compound from its origin to the movement of the solvent from the origin. The TLC plates were then sprayed with the Vanillin-sulphuric acid spray reagent (0.1 g vanillin, 28 ml methanol, 1 ml sulphuric acid) for the detection of higher alcohols, phenols, steroids and essential oils [37]. The plates were heated at 105 °C until the colours of chromatograms were optimally developed.

### Determination of minimum inhibitory concentration

The minimum inhibitory concentration (MIC) values of plant extracts against the enteric pathogens were determined using a serial dilution microplate method [38]. The test organisms used were obtained from the Enteric Diseases and Respiratory Unit of National Institute of Communicable Diseases, Johannesburg and were confirmed to be extended spectrum beta-lactamase (ESBL) positive as provided elsewhere [9]. These are *S. typhi, S. enterica* serovar Typhimurium*, Shigella flexneri* type 1b and *Sh. sonnei* phase II and typed culture of *Escherichia coli* (ATCC 25922), *Enterococcus faecalis* (ATCC 29212)*, Staphylococcus aureus* (ATCC 29213)*.*A 0.5 McFarland’s standard suspension of bacteria inoculum (1 X 10 ^8^ CFU/ml) was prepared in Mueller Hinton Broth. A series of two-fold dilutions of extracts (10 mg/ml), acetone (negative control) and gentamycin (positive control) in a microtitre plate was seeded with 100 μl of the inoculum and incubated at 37 °C for 18 h. An hour before the end of incubation, 40 μl of 0.2 mg/ml INT (*p*-iodonitrotetrazolium violet) solution was added to each well and colour development was observed at 2 h and after further incubation for 24 h. The lowest concentration where growth is inhibited was recorded as the minimum inhibitory concentration (MIC). This was indicated by a well with a decreased colour or no clear colour after incubation with INT.

### Bioautographic assay

The bioautography procedure as described by Begue and Kline [39] and refined for plant extracts by Masoko and Eloff [40] was used to identify bioactive chromatograms of plant extracts. The duplicate of TLC plates prepared were dried overnight under a stream of air to remove residual TLC solvents which may be harmful to bacteria. A 10 ml of overnight broth culture of test bacteria in Mueller Hinton broth (Merck) was centrifuged at 5300 x g for 20 min. The supernatant was discarded and the pellet was re-suspended in 2–4 ml of fresh broth and adjusted to make 0.5 McFarland standards which is equivalent to1.0 × 10^−7^ cfu/mL [41]. The dried chromatographic plates were sprayed with the test bacteria until they were completely wet in a Laminar flow cabinet (Labotec, SA). The plates were incubated overnight at 37 °C in a clean chamber at 100% relative humidity. After overnight incubation, plates were sprayed with a 2 mg/ml solution of INT (*p*-iodonitrotetrazolium violet, Sigma Chemicals). Plates were incubated and monitored for colour development at 2 h and further incubated overnight. Inhibition of growth of tested organisms was indicated by clear or yellow zones on chromatogram an indication of where reduction of INT to the coloured formazan did not take place.

## Results

### Phytochemical screening

**Fig. 1:**
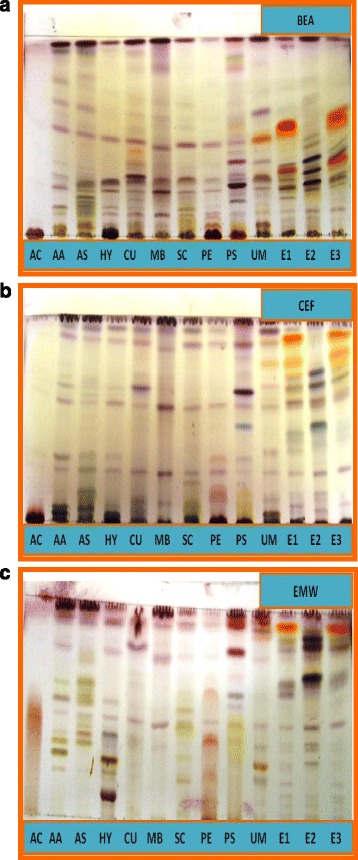
**a** TLC separation of components of the different plant extracts with BEA as eluent and vanillin-sulphuric acid spray reagent. **b** TLC separation of components of the different plant extracts with CEF as eluent and vanillin-sulphuric acid spray reagent. **c** TLC separation of components of the different plant extracts with EMW as eluent and vanillin-sulphuric acid spray reagent

The medium polar (CEF) and polar eluents (EMW) gave the best separation of compounds indicating that these extracts contained mainly relatively polar compounds. The TLC plates were photographed under short and long-wave UV light. Spraying with vanillin-sulphuric acid revealed the presence of different chemical constituents of the plant extracts indicated by the different coloured compounds (Fig. 1a–c). Characteristic green and blue fluorescence indicating presence of flavonoids according to the TLC evaluation scheme of Wagner et al. [42] were revealed under long-wave UV light (366 nm) may whereas spots of quenching of fluorescence as dark zones against light green fluorescent background with short-wavelength UV light (254 nm) indicated the presence of aromatic compounds (figures not shown).

### Bioautography assay

**Fig. 2:**
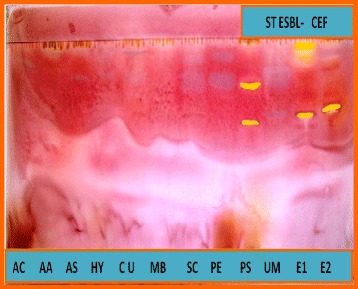
Bioautograms of components of the different plant extracts with CEF as eluent sprayed with *S. Typhimurium*

The bioautography results revealed the different compounds present in the extracts that were responsible for the antibacterial activity. *E. autumnalis, E. comosa* and *P. guajava* extracts contained one or two major bands of bioactive compounds (Fig. 2). There were two major antibacterial compounds from PS with R_f_ values of 0.64 and 0.79, two from EA with R_f_ values of 0.65 and 0.89, and one from EC with R_f_ values of 0.68 that inhibited the growth of *S. enterica* serovar Typhimurium with CEF used as the solvent system.

Active antibacterial compound was shown as clear or yellowish spot of inhibition of the growth of test organism by bioautogram. The pinkish contrasting area of bacterial growth indicated non-reduction of the tetrazolium salt by the microorganism in the presence of this compound to yield the pinkish or purplish formazan product seen in the background [39].

### Minimum inhibitory concentration

The MIC values of the plant extracts ranged from 0.018 mg/ml to 2.5 mg/ml after 24 h of incubation. The average MIC values varied for the different bacterial species with the lowest value (0.018) against *S. aureus* and *Shigella flexneri* (Table 2). Of all the crude plant extracts evaluated, *A. arborescence, C. uncinulata, E. autumnalis* and *P. guajava* had considerable antibacterial activities with MICs between 0.018 and 0.078 mg/ml. However only, *P. guajava* showed bioautograms (Fig. 2) of bioactive fractions of these plants in combination with lowest MIC. The other two plants with bioactive fractions are *E. autumnalis* and *E. comosa.* The MIC values obtained were comparable to that of the reference antibiotic (gentamicin). Of particular interest is the low MIC of *A. arborescence* against *S. aureus* and *Shigella flexneri.* The activities of *A. arborescence* and *P. guajava* against extended spectrum beta-lactamase positive *S. enterica* serovar Typhimurium is also of significance.

**Table 2:** Average Minimum Inhibitory Concentration values of plant extracts at 2 h and 24 h

Bacteria Codes	Plant Extracts and Antibiotic Codes												
	AC	AA	AS	E1	E2	HY	IQ	MB	SC	PE	PS	UM	GENT
								(mg/ml)					
EC 2H	1.25	2.5	2.5	0.625	2.5	2.5	2.5	2.5	2.5	1.25	0.156	2.5	0.039
EC 24H	1.25	0.625	0.625	0.312	0.625	2.5	1.25	2.5	2.5	1.25	0.312	1.25	0.078
EF 2H	0.625	1.25	2.5	0.312	2.5	2.5	1.25	2.5	2.5	0.312	0.078	2.5	0.625
EF 24H	0.625	0.089	0.039	0.156	0.078	2.5	0.625	2.5	0.312	0.625	0.156	0.312	0.625
SA 2H	0.312	2.5	2.5	0.078	0.312	1.25	0.625	2.5	2.5	0.156	0.078	2.5	0.078
SA 24H	0.312	0.018	0.018	0.312	0.156	0.312	0.156	0.078	0.156	0.312	0.156	0.078	0.078
SI 2H	1.25	0.156	0.312	0.156	0.312	2.5	0.625	1.25	1.25	1.25	0.312	1.25	0.625
SI 24H	1.25	1.25	0.625	1.25	2.5	2.5	2.5	2.5	2.5	2.5	2.5	2.5	2.5
ST 2H	0.039	0.078	0.312	0.078	0.078	0.625	0.625	1.25	0.312	0.312	0.156	0.625	0.156
ST 24H	0.312	0.156	1.25	0.625	0.312	2.5	1.25	2.5	1.25	0.312	0.312	1.25	1.25
STE- 2H	1.25	2.5	2.5	0.156	0.625	2.5	1.25	2.5	1.25	1.25	0.312	1.25	2.5
STE- 24H	1.25	0.078	0.078	0.156	0.625	1.25	1.25	2.5	0.625	1.25	0.312	0.625	2.5
STE+ 2H	1.25	1.25	2.5	0.156	0.312	2.5	0.625	1.25	1.25	1.25	0.078	1.25	0.078
STE+ 24H	1.25	0.078	0.312	0.312	0.625	2.5	1.25	2.5	1.25	1.25	0.312	0.625	0.156
SHF 2H	0.312	0.078	0.156	0.625	0.625	0.312	0.625	1.25	1.25	0.625	0.078	1.25	0.078
SHF 24H	0.625	0.018	0.078	0.078	0.312	1.25	1.25	2.5	0.625	0.625	0.312	0.312	0.078
SHS 2H	0.625	0.156	0.312	0.312	1.25	2.5	0.625	1.25	2.5	1.25	0.156	2.5	0.156
SHS 24H	0.625	0.039	0.039	0.039	0.312	1.25	0.312	1.25	0.625	0.625	0.312	0.312	0.156
Legend 1: Bacteria codes													
Code	Isolates												
EC 2H	*Escherichia coli* at 2 h												
EC 24H	*Escherichia coli* at 24 h												
EF 2H	*Enterococcus faecalis* at 2 h												
EF 24H	*E. faecalis* at 24 h												
SA 2H	*Staphylococcus aureus* at 2 h												
SA 24H	*S. aureus* at 24 h												
SI 2H	*Salmonella isangi* at 2 h												
SI 24H	*S. isangi* at 24 h												
ST 2H	*Salmonella Typhimurium* at 2 h												
ST 24H	*S. Typhimurium* at 24 h												
STE- 2H	*S. Typhimurium* (ESBL negative) at 2 h												
STE- 24H	*S. Typhimurium* (ESBL negative) at 24 h												
STE+ 2H	*S. Typhimurium* (ESBL positive) at 2 h												
STE+ 24H	*S. Typhimurium* (ESBL positive) at 24 h												
SHF 2H	*Shigella flexneri* at 2 h												
SHF 24H	*Sh. flexneri* at 24 h												
SHS 2H	*Sh. sonnei* at 2 h												
SHS 24H	*Sh. sonnei* at 24 h												
Legend 2: Plant codes													
Code	Plant Extract/ Control												
AC	*Acacia mearnsii*												
AA	*Aloe arborescence*												
AS	*Aloe striata*												
CU	*Cyathula uncinulata*												
E1	*Eucomis autumnalis*												
E2	*Eucomis comosa*												
HY	*Hypoxis latifolia*												
MB	*Hermbstaedtia odorata*												
SC	*Scilla nervosa*												
PE	*Pelargonium sidoides*												
PS	*Psidium guajava*												
UM	*Hydnora africana*												
GENT	Gentamycin												

## Discussion

The presence of compounds such as flavanoids and triterpenoids as indicated by the TLC plates are in accordance with some other studies and perhaps are responsible for antibacterial activities as previously described [43–45]. An active flavonoid compound, quercetin-3-O-a-l-arabinopyranoside (guaijaverin) isolated from *Psidium guajava* has been reported with high potential antiplaque agent by inhibiting the growth of *Streptococcus mutans* [46], giving credence to the use of *P. guajava* as toothpaste in folklore practices to maintain oral hygiene. Similarly, a total of seven homoisoflavonoids of varying sub-classes including a novel benzylidene type and two spirocyclic nortriterpenoids were isolated from three species of *Eucomis* by Koorbanally et al. [47]. Several reports on the biological activity of homoisoflavonoids indicated their anti-inflammatory, antibacterial, antihistaminic, antimutagenic and angioprotective qualities, and value as potent phosphodiesterase inhibitors [48, 49].

The minimum inhibitory concentration values of plant extracts against bacteria were notably lower at 24 h readings in most cases. The variations in MIC observed at 2 h and 24 h suggest that antibacterial activity of the extracts may not only be dose-dependent but also time-dependent. Whereas bacteriostatic effect may be noticeable in some plant extract-bacterial assay within 1 to 2 h some bacteriostatic or bactericidal effects are not apparent until incubation time of about 18 to 24 h. The decrease in MIC at 24 h compared with 2 h suggests that the action of the extract is bactericidal against the particular microbe as against increase in MIC overtime which is an indication of an initial bacteriostatic action of extract against the bacteria as observed for most plants against *S. enterica* serovar Typhimurium. Several studies have similarly reported time-dependent antibacterial activity of some medicinal plants [50–52]. It is surprising that there were no correlation between the MIC values and presence of antibacterial compounds after bioautography. For instance *A. arborescens* and *A. striata* had impressive MIC values but no active compounds in bioautography. This observation may be attributable to volatility, decomposition or instability of bioactive components during the course of the bioassay as previously reported [53]. For the extracts showing bioactive compounds (PS, E1 and E2), the antibacterial activity resided mostly in intermediate polarity compounds (Figs. 2a). It has been reported that bioautography allows easy localization of activity even in complex matrix as that derived from natural products [54]. Developed chromatogram comparison under identical conditions and visualized with the use of suitable chromogen reagent can provide useful information about nature of active compounds [55] and can guide isolation of active compound.

Presence of bioautogram is known to guide the chromatographic fractionation of biologically active compound, quercetin-3-O-a-l-arabinopyranoside (guaijaverin), from crude methanol extract of *P. guajava* [46]. The absence of bioautogram in some plants extracts has been adduced to instability or volatility of the bioactive chromatograms [53]. In most cases plant extracts have been reportedly shown to be more active against Gram-positive (GP) pathogens [56], a similar observation was found in this study but in addition, most of the extracts had substantial activity against the selected Gram-negative (GN) enteric bacteria as previously reported [57]. *Pelargonium sidoides* gave a moderate antibacterial activity in particular against *E. faecalis, S. aureus* and *Shigella* species. Similarly, anti-infective properties of the commercially important *Pelargonium* have been investigated [58]. *H. latifolia* did not show good antibacterial activity against most of the tested bacteria and this is in harmony with the findings on *H. decumbens* [59] even though the sterols in *Hypoxis* spp. had been reported to reduce plasma viral loads and stabilized CD4 cell counts in HIV positive patients [60], while an aqueous extract of *Hypoxis hemerocallidea (H. rooperi)* (62.5 μg/ml) inhibited some microorganisms [61].

## Conclusions

This study showed that the crude extracts of some of the herbs used in traditional medicine as remedy for stomach related ailments in our area of study have potential as antibacterial agents. *A. arborescens*, *A. striata, C. uncinulata, E. autumnalis, E. comosa* and *P. guajava* are particularly promising in the context of this study because of their bioactivities against ESBL positive bacteria and since the activities of the crude extracts compared reasonably well with gentamicin. This gives scientific credence to the use of these plants although we did not use the same extractant as traditional leaders. Also, the bioautograms are useful in bioassay-guided isolation of active compounds. Based on our results it may be useful to isolate and characterize the compounds present in *Eucomis autumnalis* and in *Psidium guajava.*

## Bacteria codes

EC 2H: *Escherichia coli* at 2h,
EC 24H: *Escherichia coli* at 24h
EF 2H: *Enterococcus faecalis* at 2h
EF 24H: *E. faecalis* at 24h
SA 2H: *Staphylococcus aureus* at 2h
SA 24H: *S. aureus* at 24h
SI 2H: *Salmonella isangi* at 2h
SI 24H: *S. isangi* at 24h
ST 2H: *Salmonella Typhimurium* at 2h
ST 24H: *S. Typhimurium* at 24h
STE- 2H: *S. Typhimurium* (ESBL negative) at 2h
STE- 24H: *S. Typhimurium* (ESBL negative) at 24h
STE+ 2H: *S. Typhimurium* (ESBL positive) at 2h
STE+ 24H: *S. Typhimurium* (ESBL positive) at 24h
SHF 2H: *Shigella flexneri* at 2h
SHF 24H: *Sh. flexneri* at 24h
SHS 2H: *Sh. sonnei* at 2h
SHS 24H: *Sh. sonnei* at 24h.

## Plant codes

AC: *Acacia mearnsii*
AA: *Aloe arborescence*
AS: *Aloe striata*
CU: *Cyathula uncinulata*
E1, E3: *Eucomis autumnalis*
E2: *Eucomis comosa*
HY: *Hypoxis latifolia*
MB: *Hermbstaedtia odorata*
PE: *Pelargonium sidoides*
PS: *Psidium guajava*
SC: *Schizocarphus nervosus*
UM: *Hydnora Africana*

## Other abbreviation

CFU/ml: *colony forming units*/ml
